# The mode of inheritance in tetraploid cut roses

**DOI:** 10.1007/s00122-012-1855-1

**Published:** 2012-04-12

**Authors:** C. F. S. Koning-Boucoiran, V. W. Gitonga, Z. Yan, O. Dolstra, C. G. van der Linden, J. van der Schoot, G. E. Uenk, K. Verlinden, M. J. M. Smulders, F. A. Krens, C. Maliepaard

**Affiliations:** 1 Wageningen University and Research Centre, Plant Breeding, P.O. Box 16, 6700 AA Wageningen, The Netherlands; 2 Present Address: Fides B.V., P. O. Box 26, 2678 ZG De Lier, The Netherlands; 3 Present Address: Horticultural Department, Henan Agricultural University, Zhangzhou City, People’s Republic of China; 4 Present Address: Sygenta Seeds B.V., P.O. Box 2, 1600 AA Enkhuizen, The Netherlands

## Abstract

Tetraploid hybrid tea roses (*Rosa hybrida*) represent most of the commercial cultivars of cut roses and form the basis for breeding programmes. Due to intensive interspecific hybridizations, modern cut roses are complex tetraploids for which the mode of inheritance is not exactly known. The segregation patterns of molecular markers in a tetraploid mapping population of 184 genotypes, an F_1_ progeny from a cross of two heterozygous parents, were investigated for disomic and tetrasomic inheritance. The possible occurrence of double reduction was studied as well. We can exclude disomic inheritance, but while our observations are more in line with a tetrasomic inheritance, we cannot exclude that there is a mixture of both inheritance modes. Two novel parental tetraploid linkage maps were constructed using markers known from literature, combined with newly generated markers. Comparison with the integrated consensus diploid map (ICM) of Spiller et al. (Theor Appl Genet 122:489–500, 2010) allowed assigning numbers to each of the linkage groups of both maps and including small linkage groups. So far, the possibility of using marker-assisted selection in breeding of tetraploid cut roses and of other species with a tetrasomic or partly tetrasomic inheritance, is still limited due to the difficulties in establishing marker-trait associations. We used these tetraploid linkage maps to determine associations between markers, two morphological traits and powdery mildew resistance. The knowledge on inheritance and marker-trait associations in tetraploid cut roses will be of direct use to cut rose breeding.

**Electronic supplementary material:**

The online version of this article (doi:10.1007/s00122-012-1855-1) contains supplementary material, which is available to authorized users.

## Introduction

Roses belong to the genus *Rosa* L. of the family of the Rosaceae, comprising about 180 species and thousands of cultivars (Debener and Linde 2009). Novel rose types with new morphological traits and colours were introduced to Europe from China during the eighteenth century, from which new groups of hybrids (Bourbon roses, Portland roses, hybrid perpetual roses and tea roses) were bred (Guoliang 2003; Joyaux 2003; Marriott 2003). A particularly interesting new group formed the tea roses obtained by crossing two of the Chinese roses with various Bourbon (*3x* & *4x*) and Noisette roses (*2x*), which were then crossed with hybrid perpetual roses (*4x*) (Zlesak 2007), in which the tetraploidy originated from *R. gallica* (*4x*). These modern roses show vigorous growth and their large flowers are borne on stiff pedicels so that they look up (Marriott 2003). Due to these intensive interspecific hybridizations, modern cut roses are complex tetraploids for which the mode of inheritance is not exactly known.

Tetraploid hybrid tea roses represent most of the commercial cultivars for cut roses currently available on the market, and they still form the basis of breeding programmes. In fact, the tea roses originate from about ten species, which is only a small part of the gene pool available for genetic improvement. Therefore, numerous other species could be used to exploit more of the genetic resources to introduce new desired traits like disease resistance. The creation of new cultivars is still mainly empirical, and new and interesting genotypes with attractive traits are fixed by vegetative propagation. If breeders want to make use of such traits in their breeding programme or if they want to enlarge the genetic basis of hybrid tea roses, a good understanding of the inheritance mode of tea rose is needed to implement an appropriately designed breeding programme. This will improve the efficiency and facilitate the transfer of novel traits into tetraploid cultivars such as disease resistances or new flower types (Byrne and Crane 2003).

Polyploidy is of importance in ornamental crops because of beneficial influences on the morphology of the plant and its organs, in particular the flower. Polyploids often also tend to be more vigorous than diploids due to the gene redundancy that masks lethal or suboptimal alleles; however, it can raise difficulties during meiosis (Comai 2005). Tetraploids can be the result of a doubling in chromosome number within a diploid species (autotetraploids), for instance obtained by fusion of unreduced gametes (Ronfort et al. 1998). In this case, all homologous chromosomes can pair during meiosis and multivalents or random pairs of bivalents can be formed, both situations resulting in a tetrasomic inheritance. Crossover events in multivalents may result in parts of two sister chromatids ending up in the same gamete: double reduction; double reduction is typical for autopolyploids forming multivalents (Ronfort et al. 1998; Stift et al. 2010).

Tetraploids can also be the result of the union of the genomes of two different diploid species and subsequent doubling of chromosomes resulting in so-called allotetraploids (Ronfort et al. 1998). If the two parental genomes are sufficiently dissimilar, then in meiosis often only pairing of homologous chromosomes occurs and not of homoeologous chromosomes, and there is no multivalent formation. In such types of tetraploids the mode of inheritance will be disomic. This is e.g., the case in octaploid strawberry (Van Dijk et al. 2012). If there is some degree of pairing between homoeologous chromosomes (Sybenga 1996), the mode of inheritance will be intermediate between disomic and tetrasomic. These polyploids may develop into forms with a strictly tetrasomic inheritance, provided that the differences in structure and gene content of homoeologous chromosomes are not too large (Stift et al. 2008). It is likely that in the original tetraploid hybrid tea cut roses there was a certain degree of preferential pairing between homologous chromosomes derived from its progenitors. After several generations of inter-crossing cut roses may have become genetically more closely related, which may mean that they now show tetrasomic inheritance, possibly for only a part of their genome, as described by Sybenga (1996). Wu et al. (1992) describe a method to estimate linkage in a segregating population of polyploids using uni-parental simplex markers. They distinguish between disomic inheritance and tetrasomic inheritance based on the frequency of detected marker pairs significantly linked in coupling or in repulsion phase. Such an analysis can contribute to a better understanding of the inheritance mode in cut roses and will be needed to get proper estimates of linkage between molecular markers as well the association between markers and for traits relevant to breeding.

Most genetic studies in rose made use of diploid mapping populations (Smulders et al. 2011) to circumvent the complexities of inheritance at the tetraploid level (Debener and Linde 2009). Another complication is skewness of the segregation of markers as shown by Byrne (2009), who reported that 10–39 % of the markers showed distorted segregation; this was ascribed to the interspecific crosses used, self-incompatibility, gametophytic selection by sub-lethal genes affecting the viability of zygote, embryo, or seedling, or maybe by competitive differences in pollen germination and pollen tube growth. These complications adversely affect the construction of molecular marker maps and genetic analyses.

The first tetraploid genetic linkage maps were published by Rajapakse et al. (2001) and Zhang et al. (2006) added genomic simple sequence repeat (SSR) markers to these maps. They based their linkage analysis on a population of 52 F_2_ plants from a cross between a tetraploid female parent with an amphidiploid male parent. Mapping resulted in two parental linkage maps, each having 14 linkage groups suggesting disomic inheritance as expected for allopolyploidy. However, these findings need not be representative of the mode of inheritance in modern cut roses because of the complex parentage. Recently, Gar et al. (2011) published a map based on a cross between tetraploid cut rose cultivars ‘Fragrant Cloud’ and ‘Golden Gate’, based on a progeny of 132 individuals. They assumed tetrasomic inheritance for their analyses.

We planned to study the inheritance in a progeny of 184 F_1_ individuals from a cross between two heterozygous tetraploid parents, both partially resistant to powdery mildew. Resistance to this disease is an important trait for breeding since powdery mildew can cause severe quality and yield losses. Assessment of resistance to this disease is difficult and time consuming; therefore, markers are seen as a good tool to facilitate selection in an early stage of a breeding programme. The possibility of marker-assisted selection for aesthetic traits like flower colour, production traits like the number of stems and disease resistances would likely lead to a gain in time to breed new varieties.

The objectives of this study were (1) to unravel the mode of inheritance by studying the segregation patterns of molecular markers in this tetraploid cross and (2) to construct two parental tetraploid genetic linkage maps by combining AFLP and SSR marker data from Yan (2005), with newly generated molecular markers, including SSR markers used in previous mapping studies.

## Materials and methods

### Mapping population and its evaluation

**Fig. 1:**
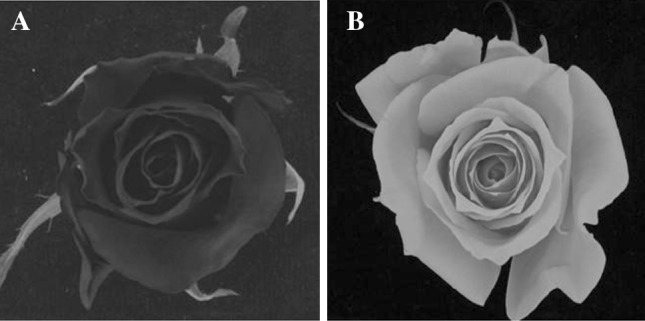
Picture of the flowers of the parents of the K5 population. **a** P540 (mother). **b** P867 (father)

The tetraploid rose population K5 from Yan (2005) investigated in this study consists of the offspring of a cross between two tetraploid genotypes P540 (mother) and P867 (father) from a cut rose hybrid tea breeding programme. P540 is a commercial cultivar developed at Terra Nigra B.V. (The Netherlands) with dark red flowers (46A, RHS colour charts, Fig. 1a). P867 has pale salmon (49C, RHS colour charts, Fig. 1b) coloured flowers and is more resistant to powdery mildew. The segregating progeny consisting of 184 genotypes was planted in a heated sun-lit greenhouse at 20/17 °C (day/night), a day length of 18 h and a relative humidity between 80 and 90 %. The experiment had a randomized complete block design with four replicates. The plants were used to determine prickle number on stems, petal number and other traits. Prickle number was assessed by counting the prickles on the main stem between nodes 4 and 6. Petals were counted when the stigmata and anthers were visible. Powdery mildew resistance data were obtained from Yan (2005) and Yan et al. (2006) who tested the K5 population for resistance against two monospore isolates (2 and F1) of *Podosphaera pannosa* (Wallr.:Fr.) de Bary (syn. *Sphaerotheca pannosa*). After inoculation with a spore suspension, development of infection symptoms was scored on a scale from 0 to 6. The scores given were 0: no symptoms; 1: very small necrotic lesions with <1 % leaf area covered with mycelium; 2: 1–5 % leaf area with mycelium; 3: 6–20 % leaf area with mycelium; 4: 21–40 % leaf area with mycelium; 5: 41–60 % leaf area with mycelium and 6: >61 % leaf area with mycelium. Disease scores were recorded 11 days after inoculation.

### AFLP marker analysis

Genomic DNA was extracted from young leaves as described by Esselink et al. (2003). AFLP markers were generated as described by Vos et al. (1995) with some minor modifications (Yan et al. 2005) using two restriction enzyme combinations, i.e., *Eco*RI/*Mse*I (E-M) and *Pst*I/*Mse*I (P-M). A prescreening for polymorphisms with different primer combinations, having either two (some *Pst*I primers) or three (some *Pst*I and all *Ec*oRI and *Mse*I primers) selective nucleotides, was done using DNA of the parents and a few individuals of the progeny. Amplified fragments of each primer-restriction enzyme combination were radioactively labelled ([γ-^33^P]-ATP), separated on 6 % denaturing polyacrylamide gels and visualized by autoradiography. Polymorphic markers were coded and dominantly scored as described in Yan et al. (2005).

### Nucleotide-binding site (NBS) profiling

NBS profiling is a multiplex screening technique, producing amplified resistance gene analogue fragments by using degenerate primers based on conserved motifs present in the NBS domain of resistance genes. NBS profiling was performed on 200 ng DNA as described in Van der Linden et al. (2004). Twelve NBS primer-restriction enzyme combinations were used to generate the NBS profiles: *Alu*I, *Hae*III, *Mse*I, and *Rsa*I combined with the degenerated primers NBS1 (5′-GTTTACTCGATTCTCAACCCGAAAG-3′), NBS3 (5′-GTWGTYTTICCYRAICCISSCATICC-3′), and NBS5a6 which is a 1:1 mixture of NBS5a (5′-YYTKRTHGTMITKGATGAYGTITGG-3′) and NBS 6 (5′-YYTKRTHGTMITKGATGATATITGG-3′). Amplified fragments of each primer-restriction enzyme combination were radioactively labelled ([γ-^33^P]-ATP), separated on 6 % denaturing polyacrylamide gels, and visualized by autoradiography.

Polymorphic bands were manually scored as dominant markers. Marker codes correspond to the first letter of the restriction enzyme followed by the number of the NBS primer and finally followed by the position of the marker on the film (e.g. *Alu*I in combination with NBS5a6 scored at position 12: A5a6-12).

### SSR marker analysis

SSR primer pairs originating from rose (Esselink et al. 2003; Süss and Schultze 2003; Zhang et al. 2006; Hibrand Saint Oyant et al. 2008), strawberry (Hadonou et al. 2004; Sargent et al. 2004; Lewers et al. 2005; Cipriani et al. 2006; Sargent et al. 2006), peach (Rajapakse et al. 2001; Dirlewanger et al. 2002), and apple (Rajapakse et al. 2001; Liebhard et al. 2002) were included in the analysis (Table 1). A PCR amplification protocol (Supplementary Material 1) was developed and optimized for each SSR primer pair. Table 1 shows which PCR protocol was used to perform amplification of the studied primer pairs. PCR amplifications were carried out on an MJ Research PTC-200 thermal cycler, in a total volume of 20 μl, using fluorescently labelled primers with the basic following profile: 30 cycles of denaturation at 94 °C for 30 s or 1 min depending on the primers, 30 s or 1 min at the primer-specific annealing temperature (Table 1) and 30 s or 1 min at 72 °C, followed by an elongation step of 10 or 30 min at 72 °C. For some primers, a touchdown process was added to the basic profile by lowering the annealing temperature from 60 to 55 °C with a step of 0.5 °C during the first 10 cycles. PCR amplified products obtained with PCR protocols 1–3, showing a clear band on an agarose gel, which were fluorescently labelled with IRDye 700 or IRDye 800, were separated by electrophoresis on a 6.5 % polyacrylamide gel using the LiCor 4300 DNA Analyzer (Westburg, The Netherlands) and manually scored using IrfanView 3.98. PCR reactions with protocol 4 were carried out on an MJ Research PTC-200 thermal cycler with the following profile: a denaturation step at 94 °C for 3 min, 30 cycles of 30 s at 94 °C, a RAMP step to reach the annealing temperature of 50 °C for 30 s and a RAMP step to reach 72 °C for 2 min, followed by an elongation step of 10 min at 72 °C. Amplified products obtained with PCR protocol 4, showing a clear band on agarose gel, which were fluorescently labelled with HEX, NED or 6-FAM, were separated by electrophoresis on a 6.5 % polyacrylamide gel using the ABI Prism 3700 DNA Analyzer (Perkin Elmer Biosystems, Foster City, Calif.). The ABI data were analysed with the Genotyper 3.6 software (Perkin Elmer Biosystems, Foster City, Calif.). All primers were obtained from Biolegio (The Netherlands).

**Table 1:** Origin of the SSR primers, PCR protocols and annealing temperatures and the results of their amplification

Species	Reference	PCR protocol^a^	Annealing temperature (°C)	Total tested	No amplification	Monomorphic	Not scorable
Rose	Esselink et al. (2003)	4	50	24^c^	5	3	6
	Süss and Schultze (2003)	3	60	130^d^	46	36	40
	Zhang et al. (2006)	1, 2 or 3^b^	50–58^b^	22^e^	6	1	0
	Hibrand Saint Oyant et al. (2008)	4	55	21^f^	3	7	2
Strawberry	Hadonou et al. (2004); Sargent et al. (2004, 2006)	4	50	28^g^	21	6	1
	Lewers et al. (2005); Cipriani et al. (2006)	4	50	24^h^	21	1	1
Peach	Rajapakse et al. (2001)	1	55 Touch down	4^i^	2	2	0
	Dirlewanger et al. (2002)	4	50	11^j^	7	0	4
Apple	Rajapakse et al. (2001); Liebhard et al. (2002)	1	55	2^k^	0	1	0

^a^Composition of the reaction mixtures for the PCR amplification protocols described in Supplementary Material 1

^b^More details are available upon request to the authors

^c^RhAB1, RhAB13, RhAB15, RhAB22, RhAB26, RhAB40, RhB19, RhB303, RhBK4, RhD201, RhD206, RhD221, RhE2b, RhE3, RhEO506, RhI402, RhJ404, RhL47, RhM405, RhO517, RhP507, RhP518, RhP519, RhP524

^d^RMS001-RMS055, RMS057, RMS058, RMS060-RMS110, RMS112-RMS116, RMS118, RMS119, RMS121, RMS123-RMS126, RMS128-RMS134, RMS137, RMS141, RMS144, RMS145

^e^Rw3K19, Rw3N19, Rw4E22, Rw5D11, Rw8B8, Rw14H21, Rw10J19, Rw10M24, Rw17I7, Rw18N19, Rw22A3, Rw22B6, Rw23H5, Rw27A11B, Rw29B1, Rw32D19, Rw45E24, Rw46O8, Rw48N6, Rw55C6, Rw61F2, Rw62C4

^f^CL2845, CL2980, Contig172, CTG21, CTG329, CTG623, H10D03, H17C12, H24D11, H2F12, Rw15D15, Rw16E19, Rw20l17, Rw23F13, Rw25J16, Rw32K24, Rw34L6, Rw52D4, Rw53O21, Rw55E12, Rw59A12

^g^EMFn018, EMFn049, EMFn110, EMFn119, EMFn121, EMFn123, EMFn136, EMFn153, EMFn160, EMFn181, EMFn202, EMFn207, EMFn213, EMFn228, EMFn235, EMFv006, EMFv016, EMFv021, EMFv023, EMFv029, EMFv104, EMFv164, EMFvi008, EMFvi018, EMFvi025, EMFvi072, EMFvi108, EMFvi136

^h^ARSFL_2, ARSFL_7, ARSFL_11, ARSFL_12, ARSFL_15, ARSFL_17, ARSFL_18, ARSFL_22, ARSFL_24, ARSFL_27, ARSFL_28, ARSFL_31, ARSFL_92, ARSFL_96, Fvi-11, UDF-002, UDF-006, UDF-016, UDF-018, UDF-019, UDF-025, UDF-033, UDF-055, UDF-065, UDF-0

^i^Pchgms3, Pchgms41, Pchcms2, Pchgms2

^j^BPPCT008, BPPCT013, BPPCT014, BPPCT017, BPPCT030, BPPCT031, BPPCT035, BPPCT037-BPPCT039, BPPCT041

^k^01a6, CH02C11

### Segregation analysis

SSRs were scored for the presence/absence of individual marker fragments, without an attempt to estimate their dosage. We use the terminology ‘phenotypic class’ when the marker genotype in terms of dosage of an allele or the parental contribution could not be observed directly. In a first analysis, only those SSR alleles were taken into account that were present in only one parent, and for which the segregation in the progeny was in agreement with a 1:1 ratio, suggesting single dosage in the parent. Segregation of SSR markers with two or three unique single-dose alleles in one of the parents was analysed in detail using the distribution of the number of individuals over the different phenotypic classes encountered in the progeny. Assuming that the markers are from a single locus, the phenotypic classes of the progeny directly reveal the allelic constitution of the gametes contributed by the parent, which allows the study of the meiosis of one parent and to investigate the mode of inheritance in that parent. The hypotheses of segregation according to disomic and tetrasomic inheritance were both tested by Chi-square goodness-of-fit test at α = 0.05. In case of allotetraploidy with a disomic inheritance, at most four phenotypic classes are expected with a frequency of 1/4 each. In case of tetrasomic inheritance with random bivalent pairing, six phenotypic classes in the progeny are expected with frequencies of 1/6 each. These six classes can be distinguished if the parent has four unique alleles at a locus that segregate in the progeny. If an SSR marker has only three different alleles that segregate from one parent, the presence or absence of the fourth allele (null allele O) can be inferred, again assuming a single-locus situation. Hence, all six possible classes can be scored. Such markers are rarely found. Therefore, also the segregation of SSR markers with two unique single-dose alleles in one of the parents of the mapping population was studied. In this situation, not all six possible phenotypic classes in case of autotetraploidy can be distinguished. Instead, four phenotypic classes are expected with frequencies 1/6, 2/6, 2/6, 1/6 for tetrasomic inheritance, and, alternatively 1/4 each for disomic inheritance. Cases of disomic inheritance with only two phenotypic classes at equal frequencies did not occur.

Double reduction is a phenomenon associated with multivalent formation in meiosis (quadrivalents, trivalents) and refers to the fact that parts of sister chromatids come together in the same gamete during the second meiotic division. The segregation data of SSR markers with three unique single-dose alleles in one parent were tested for the occurrence of double reduction. Assuming that the alleles correspond to a single locus, individuals of the progeny that displayed none of the unique alleles were assumed to have a double dose of the fourth allele (OO). Detection of a double dose of any of the three unique alleles was not possible since the marker phenotype is not different from the single-dose phenotype.

The inheritance mode was also investigated according to the procedure outlined by Wu et al. (1992). Linkage between pairs of single-dose restriction fragments (i.e., uni-parental simplex markers) was detected by calculating the χ^2^ [1] with *a*, *b*, *c*, *d* being the observed numbers of plants in the four marker genotype classes of the two loci (++, +−, −+, −−, respectively) in the progeny. χ^2^ [1] was defined as (*a*−*b*−*c* + *d*)^2^/(*a* + *b*+*c* + *d*) (Mather 1951) which was compared with the 95 %-percentile of a Chi-square distribution with one degree of freedom. For the marker pairs for which the null hypothesis (no linkage) was rejected, the linkage was estimated by estimating the recombination fraction (*r*) under the assumption of coupling phase and under the assumption of repulsion phase for disomic inheritance:

$$ \begin{gathered} {\text{Coupling, disomic and tetrasomic}}:r1 = \left( {b + c} \right)/n \hfill \\ {\text{Repulsion, disomic}}:r2 = \left( {a + d} \right)/n \hfill \\ {\text{Repulsion, tetrasomic}}:r3 = \left[ {3\left( {a + d} \right)/n} \right] - 1 \, \left( {\text{bivalents pairing at random}} \right) \hfill \\ \end{gathered} $$

where *n* = *a* + *b* + *c* + *d*.

Marker pairs were considered to be in coupling phase if *r*1 < 0.5 and in repulsion phase if *r*1 ≥ 0.5 (equivalent to *r*2 < 0.5). Under complete disomic inheritance, the expected numbers of detected coupling phase and repulsion phase linked marker pairs are equal. For the Chi-square test, the significance does not depend on which estimate of the recombination frequency is used, but just on the observed numbers of individuals in the marker classes.

For each linkage group of each parental map, the ratio between the number of coupling phase pairs and repulsion phase pairs was calculated and tested against the expected ratio 1:1 under a disomic model with a Chi-square goodness-of-fit test at α = 0.05.

### Map construction

**Fig. 2:**
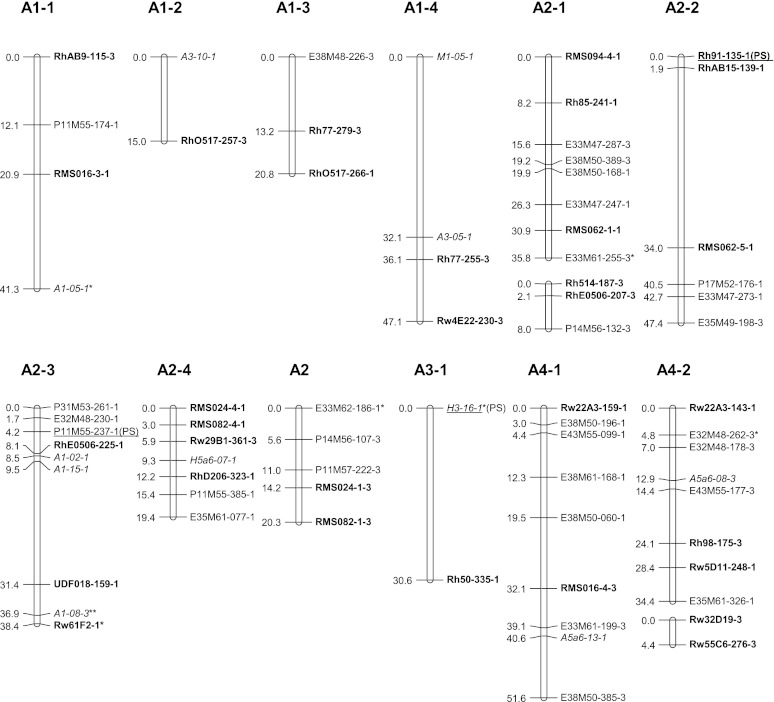

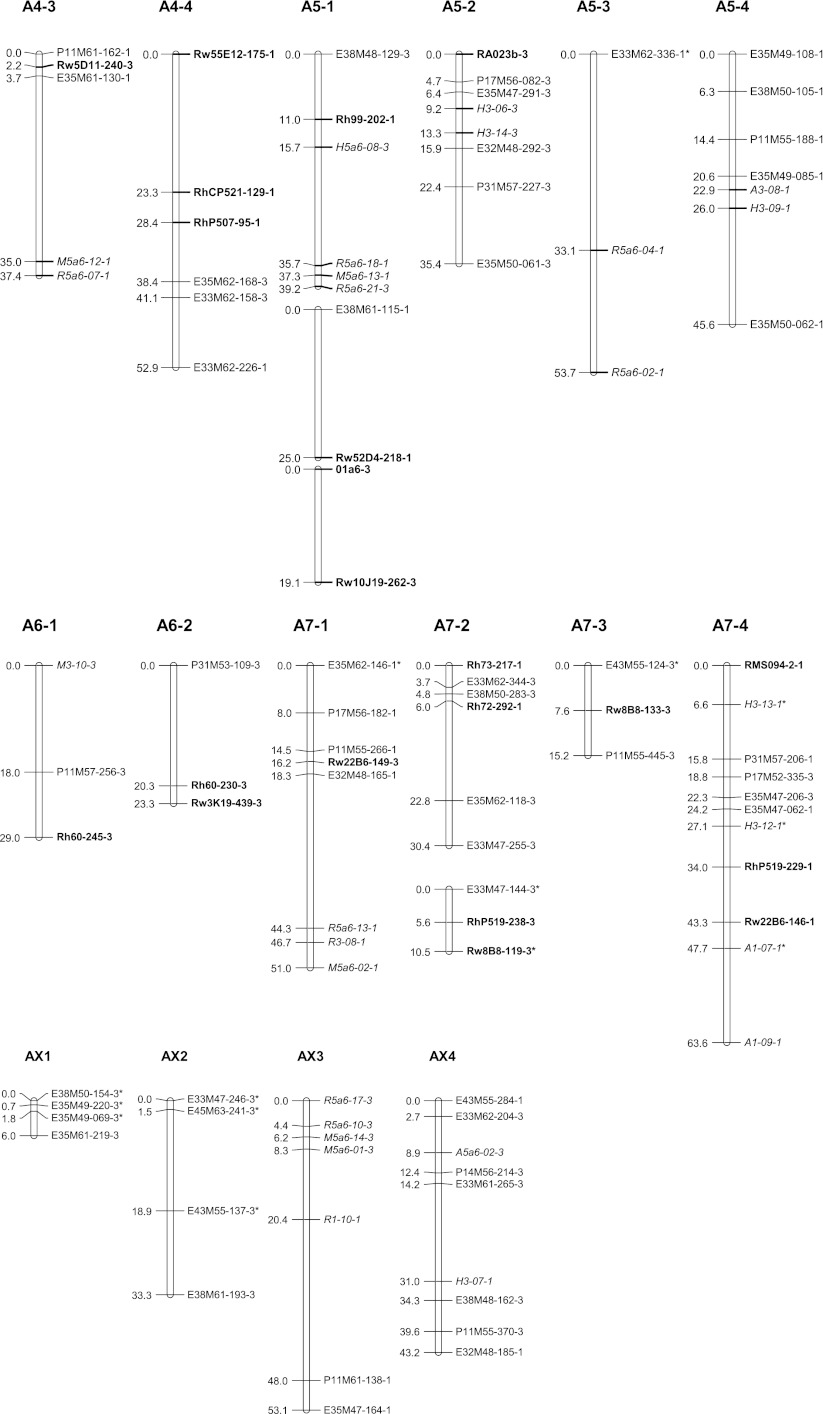
Genetic linkage map A: linkage groups of the female parent P540. Linkage groups are numbered from 1 to 7 containing each 1, 2, 3, 4 or more (parts of) homologous groups. NBS-profiling markers are highlighted in *italics* and SSR markers in *bold*. The name of uni-parental simplex markers ends with number *1*, and those of bi-parental simplex markers ends with number *3*. Markers with segregation deviating significantly at α = 0.05 from the expected ratio are marked with one asterisk. Markers deviating significantly at α = 0.01 or for which one of the parent scores was doubtful are marked with a *double asterisk*. *Underlined* markers indicate QTL positions. (*PS*) prickles on the stem

**Fig. 3:**
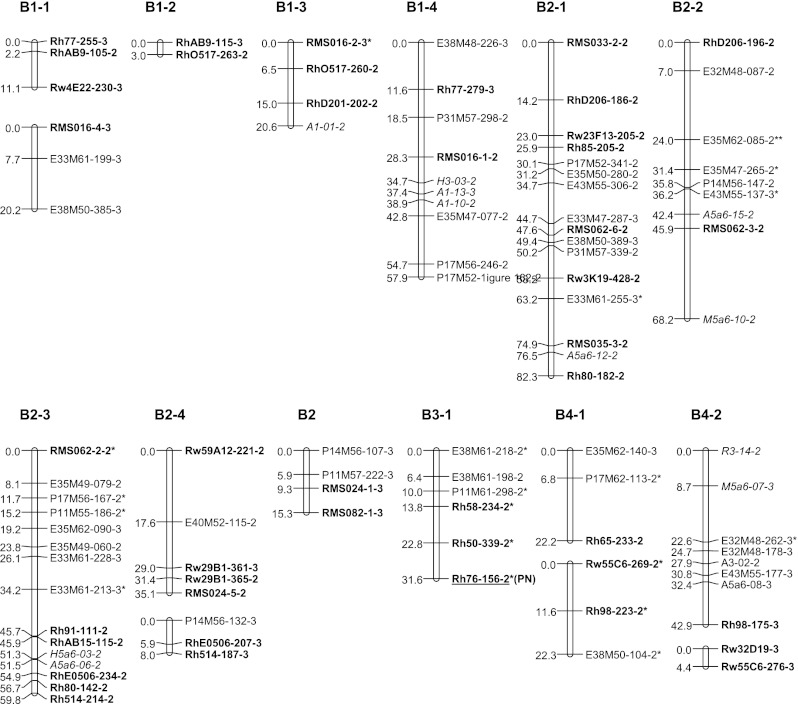

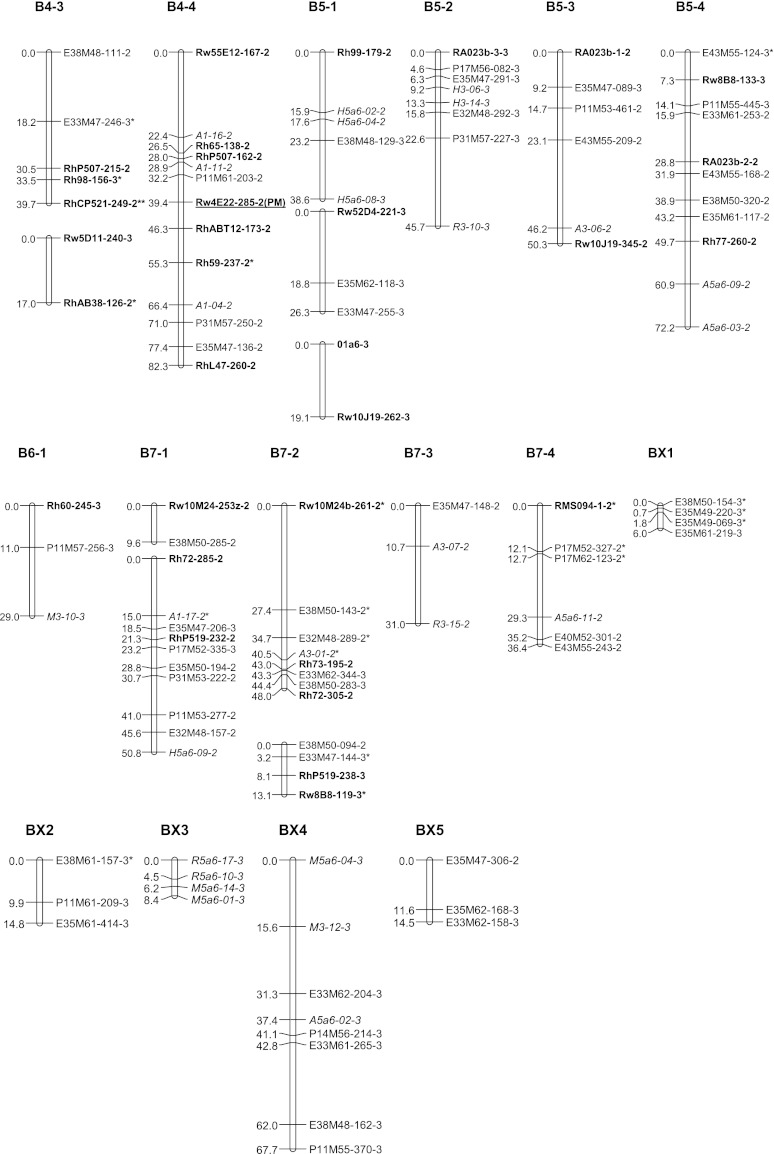
Genetic linkage map B: linkage groups of the male parent P867. Linkage groups are numbered from 1 to 7 containing each 1, 2, 3, 4 or more (parts of) homologous groups. NBS-profiling markers are highlighted in *italics* and SSR markers in *bold*. The name of uni-parental simplex markers ends with number *2*, and those of bi-parental simplex markers ends with number *3*. Markers with segregation deviating significantly at α = 0.05 from the expected ratio are marked with *one asterisk*. Markers deviating significantly at α = 0.01 or for which one of the parent scores was doubtful are marked with a *double asterisk*. *Underlined* markers indicate QTL positions. (*PN*) petal number, (*PM*) powdery mildew resistance

All polymorphic bands from NBS profiling and SSR primers were scored as presence/absence. Chi-square goodness-of-fit tests were performed on the segregation data of all markers assuming simplex segregation ratios (1:1 and 3:1). Markers deviating significantly at α = 0.05–0.01 from the ratio expected for that marker (deduced from the parent genotypes and the segregation ratio in the progeny) were included on the genetic linkage maps and marked with a single asterisk (Figs. 2, 3), whereas those with a ratio deviating significantly at α = 0.01 or ambiguous parental scores were marked with a double asterisk.

The newly generated uni- and bi-parental simplex markers were added to the tetraploid parental linkage maps of Yan (2005) using Joinmap 4.0 (Van Ooijen 2006). JoinMap does not include an option for estimating recombination frequencies in autotetraploids, but recombination frequency estimates for simplex × nulliplex markers in coupling phase are identical to those in diploids, so that the JoinMap estimates for these are valid; we preferred it over TetraploidMap as used by Gar et al. (2011) because it allows mapping of the separate coupling phase linkage groups per single chromosome. The two parental maps were constructed separately, and per parent separate linkage groups were constructed for markers in coupling phase. Linkage groups were separated using a logarithm of odds ratio (LOD) threshold of 4.0. The markers were ordered using the Kosambi mapping function. Then, sets of homologous linkage groups were identified using polymorphic SSR markers as allelic bridges, assuming that the SSR alleles are from a single locus. The resulting linkage maps were depicted with MapChart 2.2 (Voorrips 2002). Linkage groups were coded as follows: LG for linkage group followed by the number of the group and a number for the homologue (e.g., LG7-3). The last digit of each marker represents a code for the parental origin of the marker: 1 for a marker from P540, 2 for a marker from P867 and 3 for a biparental marker. These maps are based on the tetraploid parental maps of Yan (2005) and were numbered accordingly. We also aligned both parental maps to the integrated consensus map (ICM) of Spiller et al. (2010) using common SSR markers.

### QTL analysis

Phenotypic data on prickle number on stem and petal number per flower were used for marker-trait studies. This study further includes powdery mildew resistance observed by Yan (2005) and Yan et al. (2006). QTL analyses were performed with the QTL library of Genstat 14.1 using single-trait QTL analysis. Only simplex markers were included in the analysis. A genome-wide significance threshold was calculated according to the method of Li and Ji (2005) at α = 0.05. This threshold corresponds to a minus ^10^log (*p*) value of 3.127 (*p* = 0.00075). Six additional uni-parental duplex markers were tested separately by single-marker ANOVA in Genstat, using the same threshold.

## Results

### Polymorphism and segregation of the markers

Both parents and 184 offspring were genotyped for 619 markers, including those of Yan (2005). Table 2 shows the markers per type (AFLP, NBS and SSR) and according to the expected segregation ratios under disomic and tetrasomic inheritance.

**Table 2:** Classification of all markers based on the observed segregation ratios

Marker type	Marker origin	Number of markers (not significantly deviating from the expected segregation, α = 0.01)								
		Uni-parental			Bi-parental				Others^i^	Total
		S	D	D	S × S	S × D	S × D	D × D		
		1:1^c^	5:1^d^	3:1^e^	3:1^c^	7:1^e^	11:1^d^	35:1^d^		
AFLP^a^	P540	48	16						4	68
	P867	56	9						12	77
	P540 and P867				68	5	22	6	6	107
NBS	P540	49	3	2					5	59
	P867	56	4	1					7	68
	P540 and P867				26^f^	1	5^g^		9	41
SSR^b^	P540	46	5	2					1	53
	P867	82	6						4	92
	P540 and P867				36^f^		7^h^	7	3	54
	Total	337	43	5	130	6	34	13	51	619

*S* simplex, *D* duplex

^a^Yan (2005)

^b^Part of the data from Yan (2005)

^c^Expected ratio in case of disomic or tetrasomic inheritance

^d^Expected ratio in case of tetrasomic inheritance

^e^Expected ratio in case of disomic inheritance

^f^The segregation of one marker was not significantly different from 7:1 either

^g^The segregation of two markers was not significantly different from 7:1 either

^h^The segregation of four markers was not significantly different from 7:1 either

^i^Skewed segregation (α = 0.01)

Twenty-six AFLP primer pairs (Yan 2005) generated 252 polymorphic markers of which 172 (68 %) were considered simplex as segregation was in agreement with (not significantly different from) either a 1:1 or a 3:1 segregation (Table 2). Fifty-three markers (21.0 %) were considered duplex as not significantly different from either 5:1 (duplex × nulliplex), 11:1 (duplex × simplex) or a 35:1 (duplex × duplex) segregation (Table 2).

From the NBS gels, 168 polymorphic markers were dominantly scored with a maximum of 24 polymorphic markers per combination (for NBS5a6-*Rsa*1). Table 2 shows that 132 (79 %) of the NBS markers were considered simplex in one or both parents (segregation in agreement with a 1:1 or 3:1) while 12 markers (7.1 %) were considered duplex in one or both parents (5:1, 11:1 or 35:1; Table 2).

SSR markers available from the literature for Rosaceae were used to expand both parental maps and to allow alignment to other existing maps. Out of 197 primer pairs developed for rose, 137 (70 %) amplified well and showed clear bands on agarose gel (Table 1). The parents were polymorphic for 42 of these SSR markers (31 %). From the strawberry SSRs tested, only 9 out of 52 (17 %) showed a clear amplification on agarose gel. Only a single primer pair (UDF-018) showed a polymorphism between the parents (Table 1), resulting in one simplex allele and two alleles (simplex × duplex) segregating 11:1 (α = 0.01). Only the simplex allele was included in the construction of the genetic maps and was mapped. Two peach primers (Pchcms2 and Pchgms2) out of 15 tested gave amplification but the PCR products were monomorphic (Table 1). Finally, one of two apple SSR primers (01a6) gave an amplified fragment (Table 1) and one bi-parental simplex marker. Out of the 199 amplified SSR markers, 164 (82.5 %) were considered simplex (1:1 or 3:1 segregation) while 25 (12.6 %) were considered duplex (5:1, 11:1 or 35:1 segregation). The presence of the latter segregation ratios for duplex markers is indicative of tetrasomic segregation (Table 2).

Five markers out of the set of uni-parental markers were considered as duplex segregating in agreement with a 3:1 type of segregation. Three of them could be mapped on different linkage groups. Moreover, six markers were considered as simplex–duplex with a 7:1 segregation ratio (Table 2). Such segregation ratios are indicative of disomic inheritance.

Eight markers not significantly deviating from duplex segregation ratios as expected in tetrasomic inheritance were also not significantly different from a 7:1 segregation ratio (Table 2), which is typical of a disomic inheritance mode. None of the markers with duplex condition in the parents were found to segregate in accordance to 15:1 ratio as expected in case of disomic inheritance. These results may suggest an inheritance mode with a certain degree of preferential pairing as in Stift et al. (2008).

The hypothesis of some preferential pairing of chromosomes was further investigated using markers for which one single parent has a single dose for two or three marker alleles. Six phenotypic classes are expected for markers with three single-dose alleles in case of tetrasomic inheritance if they belong to the same locus. Only four are expected in case of disomic inheritance. The hypothesis of tetrasomic inheritance (each class expected to have a frequency equal to 1/6) was tested with a Chi-square goodness-of-fit test (α = 0.05, *df* = 5) for the uni-parental three-allelic marker RMS033 from parent P867. Offspring plants for this marker exhibited patterns in the six predicted phenotypic classes in a ratio not significantly different from expectations for tetrasomic inheritance (Table 3). From the SSR markers with alleles present only in parent P540 none exhibited more than two segregating alleles in the progeny.

**Table 3:** Distribution of gametic contribution to genotypes from the mapping population for the triplex P867 SSR marker RMS033 (ABCO × OOOO)

Marker	Gametic contribution								Gamete unknown	χ^2^ value	Linkage groups
	AB	BC	AC	AO	BO	CO	OO	ABC		Tetrasomic inheritance	
RMS033	21	13	19	20	20	31	14	14	23	0.146^a^	B2-1

Chi-square goodness-of-fit test (χ^2^ at *df* = 5, *n* = 124) values to assess a segregation different from 1:1:1:1:1:1 for gametes AB, BC, AC, AO, BO and CO, O being a null allele, and OO and ABC being unexpected phenotypes

^a ^No significant difference from a 1:1:1:1:1:1 segregation ratio

SSR markers showing segregation for two unique alleles derived from one parent were also tested to get more insight into the mode of inheritance. In this situation, only four marker classes are possible, but it is possible to test the inheritance pattern, considering that the four phenotypic classes have expected frequencies of 1/6, 2/6, 2/6, and 1/6 in case of tetrasomic inheritance (random pairing of bivalents or quadrivalent formation but without double reduction) and 1/4 each in case of disomic inheritance. Here also, a Chi-square goodness-of-fit test was used (χ_0.95_^2^ = 7.82, *df* = 3, Table 4). Frequencies of the phenotypic classes of the progeny for two of the markers tested (RhD206 and RhP507 for parent P867) were consistent with the hypothesis of tetrasomic inheritance (Table 4). For Rh65, the hypothesis of disomic inheritance was not rejected (Table 4). For Rw22A3 (from P540), neither the hypothesis of disomic inheritance nor the hypothesis of tetrasomic inheritance was rejected (χ_0.95_^2^ = 7.82, *df* = 3, Table 4). For marker Rh99 both hypotheses were rejected (Table 4).

**Table 4:** Distributions of gametic contribution of parents P540 and P867 to the mapping population for various SSR markers

Parent	Marker	Gametic contribution				Gamete unknown	χ^2^ value		Linkage groups
		AB	AO	BO	OO		Tetrasomic inheritance	Disomic inheritance	
P540	Rw22A3	37	49	49	29	11	4.70^a^	7.02^a^	A4-1/A4-2
	Rh99	33	54	45	43	0	10.43^b^	18.4^b^	A5-1
P867	RhD206	21	51	38	31	34	4.72^a^	13.52^b^	B2-1/B2-2
	Rh65	46	50	45	33	1	16.64^b^	4.24^a^	B4-1/B4-4
	RhP507	30	52	35	30	28	6.65^a^	8.89^b^	B4-3/B4-4

The segregation was tested using a Chi-square goodness-of-fit test (χ^2^, *df* = 3) assuming segregation ratios for either tetrasomic or disomic inheritance

^a^No significant difference from expectation

^b^Null hypothesis (disomic or tetrasomic inheritance) rejected at α = 0.05

### Double reduction and meiotic irregularities

The SSR uni-parental marker RMS033 (with three unique alleles) showed allelic combinations and a segregation pattern in agreement with tetrasomic inheritance (Table 3); however, 8 % of the progeny displayed none of the visible alleles present in the parent P867, whereas two would be expected if segregating from a single locus (Table 3). This phenotypic class with a double dose of the null allele (OO) can be explained by the phenomenon of double reduction or by assuming that the SSR fragments are not from a single locus. The percentages mentioned above cannot be taken as estimates for the total amount of double reduction: phenotypic classes AO, BO and CO could contain double dosage of the A, B or C allele, respectively, which would also be products of double reduction, but which cannot be distinguished from single dosage of the visible allele. This marker is located at the end of its linkage group where the probability for double reduction to occur would be higher in case this is the distal end of the chromosome away from the centromere.

For most of the SSR markers, simplex and duplex alleles were present in both parents, which allowed studying the inheritance pattern by counting the different phenotypic classes displayed by the progeny. Not all phenotypic classes were always distinguishable; nevertheless, it was possible to investigate whether alleles are inherited together or not. A total of 11 inheritance patterns were further investigated for SSRs displaying a mix of simplex and duplex alleles (Supplementary Material 2, supplies the details of all the observed inheritance patterns). For all SSRs, all phenotypic classes expected in case of tetrasomic inheritance were present in the progeny but their frequencies were not always as expected. Besides, for RhCP521, the phenotypic classes OOOO and DOOO were observed for 39 individuals (22.3 %, Supplementary Material 2). Those classes are only possible in case of double reduction. The phenotypic class (OOOO) was also observed for seven individuals (with two individuals common to both markers) of a nearby marker Rh98 (6.2 cM). Linkage groups B4 contain five markers (RhCP521, Rh65, Rh98, RhL47, Rw55E12) that show phenotypic classes that can be explained by occurrence of double reduction.

For RMS082, where two models are possible (BCDO × ABCC or BCCD × ABCO, Supplementary Material 2), gametes BO and DO are not found and the phenotypic class ABCD is three times larger than expected, which suggest preferential pairing for this SSR. The phenotypic class ABCDE for RMS094 (ABCO × CDEO) was observed for eight (5.2 %) individuals of the progeny (Supplementary Material 2). Such a phenotype can only be explained if three alleles of one parent are transmitted to the progeny, which theoretically is not possible. Possibly the primers amplify a fragment at another locus in the genome, or two SSR loci on different locations. In the case of RMS094, five fragments were amplified from the two parents but only three fragments were mapped, one for each parent in linkage group 7 (A7-4 and B7-4) and one in linkage group A2-1. This indicates that here a second locus is amplified as well.

### Mode of inheritance

Two ways of calculating recombination frequencies between pairs of markers were used. One set of estimates for coupling phase simplex × nulliplex and simplex × simplex marker pairs was calculated with JoinMap in order to generate linkage maps. JoinMap does not take into account tetrasomic inheritance, but estimates for simplex × nulliplex markers in tetraploids are identical to those of coupling phase markers in a diploid.

Pairwise estimates of recombination frequencies were also calculated under both a disomic and a tetrasomic model according to a procedure of Wu et al. (1992). They used a χ^2^[1] test to determine significance of linkage in polyploids. The pairwise recombination fractions obtained with this method under the assumption of coupling phase (*r*1) and under the assumption of repulsion phase (*r*2, *r*3) allowed validation of the assignment of the linkage phases in the genetic linkage maps constructed with JoinMap since the method of Wu et al. (1992) takes tetraploid segregation (i.e., tetrasomic inheritance with random pairing of bivalents) into account. In autotetraploids there are three possibilities for random pairing of two sets of two bivalents. The calculations gave an argument to assign AX3 to linkage group A2-1 since three investigated uni-parental markers located on AX3 were in coupling phase with four of the uni-parental markers of the linkage group A2-1, and in repulsion phase with two uni-parental markers from linkage group A2-2, and with all eight uni-parental markers of linkage group A2-3. This confirmed the grouping of markers as obtained by JoinMap.

The numbers of marker pairs that are significantly linked in coupling phase and in repulsion phase were calculated for each set of linkage groups (per chromosome per parent), and a Chi-square goodness-of-fit test was performed (α = 0.05) to test the hypothesis of a 1:1 ratio coupling: repulsion for the numbers of pairs per linkage group, which would correspond to disomic inheritance (Table 5). For five linkage groups of 11 investigated (Table 5), the hypothesis of a 1:1 ratio was rejected, indicating that for those linkage groups there is no complete preferential pairing according to a disomic inheritance. The SSR markers on most of these linkage groups show phenotypic classes expected for tetrasomic inheritance. For six linkage groups, the 1:1 ratio for coupling/repulsion marker pairs was not rejected, so the numbers of coupling/repulsion phase pairs are in agreement with the expectation under a disomic mode of inheritance (Table 5). On closer inspection, however, we noticed that the distribution of the recombination frequency estimates under repulsion under the assumption of a disomic inheritance is skewed toward higher values and that the significance levels are lower than for the coupling phase pairs of markers. We also considered that the significance threshold of α = 0.05 is not strict enough, considering the large number of marker pairs that we are considering. Therefore, we repeated the analysis with a stricter threshold and used the LOD score for independence (as calculated in JoinMap) with a threshold of 3.0 as the significance criterion (results not shown). If we then compare the numbers of marker pairs in coupling phase and repulsion phase, we clearly see that for all linkage groups, we obtain much larger numbers of significant pairs in coupling phase than in repulsion phase. The ratios of these numbers per chromosome are significantly different from 1:1 (results not shown). These observations fit much better with a tetrasomic inheritance or possibly a combination of disomic and tetrasomic inheritance.

**Table 5:** Chi-square goodness-of-fit test at α = 0.05 to test the hypothesis of a 1:1 ratio coupling: repulsion per linkage group

Linkage group	χ^2^ *df* = 1	*N*
A1	0.44^a^	36
A2	0.07^a^	232
A4	8.36^b^	115
A5	12.23^b^	106
A7	11.33^b^	195
B1	0.10^a^	91
B2	0.24^a^	697
B3	9.66^b^	70
B4	19.43^b^	245
B5	0.68^a^	212
B7	0.03^a^	323

*N* number of coupling- and repulsion-phase-linked marker pairs

^a^No significant difference from expectation

^b^The 1:1 hypothesis was rejected

### Parental linkage maps

A total of 275 markers was used to construct the parental linkage map of P540 (Map A, Fig. 2), comprising 143 uni-parental simplex markers and 132 bi-parental simplex markers. This parental map contains 172 loci over 28 linkage groups covering 1,081 cM. A set of 326 markers was used to construct the parental linkage map of P867 (Map B, Fig. 3), comprising 194 uni-parental simplex markers and 132 bi-parental simplex markers. This map contains 209 loci distributed over 30 linkage groups spanning 1,225 cM. Each map is expected to have a total of 28 linkage groups per parental map, corresponding to the seven chromosomes times four coupling phase linkage groups (homologs/homoeologs) per chromosome. A small number of linkage groups could not be assigned yet. The linkage groups obtained so far comprise all chromosomes, but they do not all come in sets of four per parent per chromosome. The situation for chromosomes A1, B1, A4, B4, A5, B5, A7and B7 meets the expectation, whereas the A2 and B2 sets contain an extra linkage group and the A3 and B3 sets so far contain only one linkage group. The 6A set derived from P540 consists of two linkage groups and the B6 set from P867 of only one. Four linkage groups from P540 (AX1, AX2, AX3 and AX4), and five linkage groups from P867 (BX1, BX2, BX3, BX4, BX5) could not be assigned to any of the chromosomal linkage groups yet since they do not contain SSR markers with known map position (Figs. 2, 3). Most markers in these groups were dominantly scored simplex bi-parental markers segregating in a 3:1 ratio. Estimates of recombination frequencies between such markers tend to have a large error and low significance. This is probably the reason that they could not be integrated into any of the other linkage groups. Only AX3 might be moved to linkage group A2-1 since there are some fairly high LOD scores for markers between these two groups. The recombination data of simplex × nulliplex markers as obtained with the approach of Wu et al. (1992) point in the same direction.

Multi-allelic SSR markers were successfully used to assign linkage groups to the seven basic sets of linkage groups (Figs. 2, 3). Some of the short linkage groups could also be assigned to the LG groups (sets of linkage groups for the same chromosome) because they contained SSR loci which were either present on the tetraploid maps of Yan (2005) or on the ICM of Spiller et al. (2010). Linkage groups of LG1 were identified by SSR markers RMS016 and Rh77. Other SSR markers RhAB9, RhO517 and RhD201 confirmed this assignment since they were mapped to LG1 of the ICM as well. However, there are some differences between our linkage maps and the diploid ICM. Two alleles of Rh77 were assigned to A1-3 and A1-4 (Fig. 2), and to B1-1 and B1-4 (Fig. 3), but another marker from this SSR was mapped to linkage group 5 on the diploid ICM. Possibly the latter fragment corresponds to another locus. Furthermore, a bi-parental allele of marker Rw4E22 was mapped to linkage groups B1-1 and A1-4, whereas it was mapped to linkage group B4-4 for a uni-parental allele (Figs. 1, 2).

The multi-allelic markers Rh514, RhD206, RhE0506, RMS024, RMS082 and Rw29B1 enabled us to identify on both parental maps groups homologous to LG2. Markers RhAB15, Rh80, Rh85, Rh91, RMS062 and Rw59A12 confirmed this assignment since they were mapped to LG2 on the ICM as well. Nevertheless, for both maps five groups instead of the expected four make up this linkage group. This is probably due to a lack of a sufficient number of anchoring markers. Marker RMS035 was mapped to group B2-1 whereas it was mapped to linkage group 7 on the diploid ICM. This marker is closely linked to the marker Rh80, which is also assigned to LG2 in the ICM. There could be a duplication of this locus since it was also mapped to LG2 in one of the ICM populations.

The coverage of LG3 is very low since only one group of coupling phase markers could be identified. This group contains the marker Rh50 (mapped in both parents) and Rh76 for P867 which are also mapped to LG3 in the diploid ICM.

The multi-allelic markers RhP507, RhCP521 and Rw5D11 enabled us to identify LG4 groups for both parents. This assignment was confirmed by markers Rh59, Rh65, Rh98, Rw55C6 and Rw55E12 because these were mapped to LG4 in the ICM as well. Two alleles of marker Rw22A3 were mapped to two subgroups of LG4, whereas they were mapped to LG6 on the diploid ICM. One of the allele was assigned to A4-2 on our maps because it shows a high LOD score (7.65) with Rw5D11, which is 0.3 cM from Rh98 that mapped on LG4 on the diploid ICM. Marker RhAB38 was assigned to B4-3 whereas in the diploid ICM, it was mapped on LG5.

The multi-allelic marker RA023b enabled us to identify linkage groups of LG5 for both parents. Other groups could be assigned to this LG set from the markers Rh77, Rh99, Rw10J19 and Rw52D4 as in the diploid ICM.

Linkage group 6 contains two marker groups for P540 (A6-1 and A6-2, Fig. 2) and one for P867 (B6-1, Fig. 3). The anchoring marker Rh60 is mapped to LG6 of the ICM as well. One 3:1 segregating marker from Rw3K19 was mapped to A6-2 for P540, but a second one, segregating 1:1, was mapped to B2-1 for P867. The multi-fragment markers Rh72, Rh73, RhP519, Rw10M24 and Rw22B6 allowed to identify three marker groups of LG7 (Figs. 1, 2). These were mapped to LG7 of the ICM as well. The markers RMS094 and Rw8B8 were used as anchoring markers between both parental maps. Rw8B8 is strongly linked to RhP519, which is mapped to LG7 of the diploid ICM. However, Rw8B8 was assigned to LG5 on the diploid ICM as we did on B5-4 for another fragment amplified by this SSR. This suggests that this SSR amplifies several loci from which one is similar to the one amplified for the ICM.

### QTL mapping

Identified QT Ls for prickles on the stem, petal number and powdery mildew resistance are shown in Table 6 and indicated on the linkage maps (Figs. 1, 2). Three markers (Rh91-135, P11M55-237, H3-16) together explain 44 % of the variance of the number of prickles on the stem, which is about half of the heritability (Yan et al. 2006; Table 6). Rh76-156 explains 13 % of the variance for petal number. Marker Rw4E22-285 explains 8.5 % of the variance of powdery mildew resistance. None of the duplex markers had a significant association with the traits studied.

**Table 6:** QTLs identified for three traits

Trait	Marker	LG	−^10^log (*p* value)	% variance explained	Heritability^a^ (*h* ^2^)
Prickles on the stem	Rh91-135	A2-2	6.1	13.1	0.90
Prickles on the stem	P11M55-237	A2-3	4.1	8.1	0.90
Prickles on the stem	H3-16	A3-1	9.5	22.9	0.90
Petal number	Rh76-156	B3-1	3.7	12.7	0.88
Powdery mildew resistance (isolate F1)	Rw4E22-285	B4-4	3.3	8.5	0.62

^a^Heritability (*h* ^2^) after Yan et al. (2006)

## Discussion

### Assessment of the parental maps

The length of the parental tetraploid maps was 1,081.3 cM for P540 (172 loci) and 1,225.4 cM (209 loci) for P867. Yan et al. (2005) estimated the expected length of the diploid rose map to be 500 cM using repeated sampling without replacement of marker pairs from the parental maps (Stam, unpublished results). For a tetraploid map with four linkage groups per chromosome, this would correspond to 2000 cM per parent. This suggests that the maps in this study cover approximately 54–61 % of the estimated expected length.

Yan’s tetraploid parental maps (2005) covered 695 cM for P540 (102 markers) and 697 cM for P867 (110 markers), which correspond to about 35 % coverage. The number of groups per chromosome has been improved considerably since Yan’s maps did not contain a complete set of four groups for any of the chromosomes.

Alignment of individual diploid genetic linkage maps has been attempted within several mapping projects (Debener and Linde 2009). As a result, Spiller et al. (2010) published the first integrated consensus diploid genetic linkage map (ICM) for rose, based on four diploid genetic maps. They used the same numbering for the linkage groups as Yan et al. (2005) and as we did in this study. This enabled the comparison across published maps and helped to infer the assignment of small linkage groups (A4-2, A5-1, A7-2, B4-1, B5-1, B7-1, B7-2) to linkage groups.

The differences that remain between the genetic maps might be due to differences in transferability of the SSR markers between all populations. They may also be the result of the occurrence of multiple loci amplified with a single primer pair, in combination with segregation of different fragments in the populations studied. Similar to the diploid ICM, in our maps linkage groups 3 and 6 were the least covered. For both parental maps they consisted of one group of markers only instead of the expected four. Spiller et al. (2010) found that markers of linkage group 3 showed skewed segregation (we observed this also in linkage group B3-1) and explained this by the action of a gametophytic self-incompatibility locus on linkage group 3. As for linkage group 6, there were too few markers to anchor individual linkage groups, which could be explained by a low recombination rate for this linkage group as shown in Spiller et al. (2010). In addition, the markers were not very informative as they were all dominant bi-parental simplex markers with 3:1 segregation.

We could identify one of the suggested linkage groups of the rose diploid map of Zhang et al. (2006), based on three markers common to our maps: Rw8B8, Rw10M24 and Rw22B6. Zhang et al. (2006) placed them on their consensus map in one linkage group, which corresponds to linkage group 7 of our maps. The fragments amplified by this SSR correspond probably to another locus than the one amplified by Rw8B8 and assigned on LG5 by Spiller et al. (2010) and on FC5 by Gar et al. (2011), confirming the multi-loci feature of this SSR. Unfortunately, it was impossible to establish more correspondences to their map due to a lack of common SSR markers.

Gar et al. (2011) published integrated genetic linkage maps of a tetraploid rose population of 132 tetraploid individuals. They used the software TetraploidMap to construct their linkage maps. TetraploidMap has the advantage to be able to include uni-parental duplex markers, but cannot cope with markers showing double reduction, and the linkage phase of the markers has to be manually inferred. They constructed two genetic linkage maps composed of seven linkage groups, which combine all markers present in the four groups per chromosome. To construct our linkage maps, we used the software JoinMap using only simplex markers. The linkage maps generated in this way showed all homologous groups separately, which will be useful for QTL mapping. We also investigated the mode of inheritance and think that, in our population, it might be more complex than the complete tetrasomic inheritance as assumed by Gar et al. (2011) in their population. The markers in common between both maps show the same correspondence as was found with the diploid ICM. FC1 and GG1 correspond to our LG3. FC2 and GG2 correspond to our LG2 except for marker Rh98, which was assigned to LG4 in our maps as well as in the diploid ICM. FC3 and GG3 correspond to LG1. FC4 and GG4 correspond to LG7 except for marker Rw59A12 which was assigned to LG2 in our maps as well as in the ICM; marker Rw8B8 was assigned to LG7 in our maps while it was on LG5 on the ICM. FC5 and GG5 correspond to LG5 except for RhAB38 which was assigned to LG B4. FC6 and GG6 correspond to LG4 and FC7 and GG7 correspond to LG6 of our maps.

Several QTLs were identified for number of prickles on the stem. They were located not only on linkage groups A2-2, A2-3 but also on A3-1. One QTL was identified for petal number located on linkage group B3-1. Two of our QTLs are in accordance with QTLs for prickles and petal number identified by Spiller et al. (2010) and which they mapped on LG3. Several QTLs for resistance to powdery mildew were identified on the diploid ICM. Among these are two regions on LG4, where we also identified a QTL for powdery mildew resistance. However, the tetraploid map of Gar et al. (2011) shows the marker PM2 responsible for powdery mildew resistance mapped in FC7 (corresponding to our LG6). This demonstrates the polygenic nature of powdery mildew resistance.

### Comparison of SSRs among Rosaceae

This study also provided a way to study the diversity at SSR loci between cut rose and other representatives of the Rosaceae family. Large differences in amplification and degree of polymorphism were observed among the primers used. Seventeen of the 22 tested SSR primers developed by Zhang et al. (2006) showed excellent amplification and polymorphism (Table 1). These primers were developed from a genomic library of the diploid *R. wichurana* ‘Basye’s Thornless’. These primers also gave a high percentage of reliable amplification products when screening the tetraploid 90–69 mapping progeny of Rajapakse et al. (2001), (70 % of 43 tested primers) and the population of Hibrand Saint Oyant et al. (2008), (94 % of 16 tested primers). In addition, 76 % of the SSR markers from Hibrand Saint Oyant et al. (2008) showed amplification in our population (Table 1). These primers were developed for a cross in which one of the parents was a dihaploid obtained from haploidisation from a tetraploid *Rosa hybrida* cv Zambra. SSR primers developed by Esselink et al. (2003) on cut rose showed 54 % amplification (Table 1).

In contrast to Hibrand Saint Oyant et al. (2008), we had very little success in amplification (2 vs. 41 %) with strawberry primers (Table 1). The same was true for peach SSR primers from which no marker could be produced for our population, whereas Zhang et al. (2006) were able to map some of them (pchgms2 and pchgms3) and use them for comparative mapping since they are located on the *Prunus* reference map. Only one of the four markers obtained from the primer pair UDF018 from strawberry turned out to be present in simplex condition in P540 and was mapped on the corresponding parental map (A2-3, Fig. 2). Moreover, 01a6 from apple produced one polymorphic marker segregating 3:1 (simplex–simplex) and was mapped in both parental maps to a small linkage group containing two markers, which could be manually linked to the linkage group B5-1 by aligning the SSR markers to the ICM. The success rate of amplification of the SSRs was clearly higher for SSRs originating from roses than from other Rosaceae (ca. 70 vs. 26 %, Table 1). Only 34 % of the rose SSRs were monomorphic, demonstrating that our parents are representative for the genetic material used in breeding programmes.

### Inheritance mode

Our results showed that RMS033 with three unique alleles of a single parent was not compatible with a disomic type of inheritance if we assume that it corresponds to a single locus (Table 3). Various other markers were also not compatible with a disomic type of inheritance (Table 4). For Rh99 strict tetrasomic inheritance was also rejected (Table 4). From the analyses of the pairs of uni-parental simplex markers according to Wu et al. (1992), but using a strict criterion based on the LOD score for independence, the hypothesis of complete disomic inheritance was rejected, suggesting a tetrasomic inheritance or an inheritance with some but not complete preferential pairing of chromosomes. Stift et al. (2008) inferred an inheritance mode intermediate to disomic and tetrasomic inheritance for yellow cress, especially for fertile interspecific hybrids. It is possible that this situation occurs in our cut rose population as well. Sybenga (1996) described a phenomenon where some chromosomes pair preferentially with homologs, while others also readily pair with homoeologous chromosomes. Such types are called segmental allotetraploids. If there is some pairing between homoeologous chromosomes, this would result, after several generations of recombination, in a loss of preferential chromosome pairing and subsequently in the establishment of true autopolyploids with tetrasomic inheritance for all chromosomes.

Evidence for the occurrence of double reduction comes only from the inferred presence of a double dose of null alleles and under the assumption that the other unique alleles correspond to a single segregating locus for each of the SSR loci. The segregation ratios of the other alleles support that assumption. However, from the comparison with other maps we concluded that some SSRs may actually correspond to multiple loci. Also, not all alleles of the SSRs from which the evidence for double reduction was deduced could be mapped. Therefore, clearly more markers will be needed for proof for the occurrence of double reduction.

Ours proved to be an ideal population to study the genetics of agronomic traits of cut roses and to gain knowledge that meets the breeder’s needs. It is highly heterozygous like most cut rose cultivars used in breeding programmes and segregates for many commercially important traits such as flower production and disease resistance. The integration of knowledge about the inheritance mode and segregation at marker loci at a tetraploid level, and the association between markers and traits, will help to direct the breeding programme to develop genotypes combining multiple traits of interest to breeders. Moreover, the rose genome sequence might become available shortly, which will make it easier to specifically design markers, for instance by homology search with other existing functional markers, and cheaper due the massive generation of SNP markers. This will allow the number of markers to be increased dramatically, which, in turn, will enable to study in more detail the tetraploid inheritance of markers and quantitative traits in our population and in other tetraploid commercial crosses.

## Electronic supplementary material

Below is the link to the electronic supplementary material.

Supplementary material 1 (DOC 32 kb)

Supplementary material 2 (XLS 76 kb)
