# Supplementary material for: The mode of inheritance in tetraploid cut roses
Source: Theor Appl Genet. 2012 Apr 12;125(3):591–607. doi: 10.1007/s00122-012-1855-1 (PMC3397129; doi:10.1007/s00122-012-1855-1)
Supplement: Supplementary file 1 — Supplementary material 1 (DOC 32 kb) [file 122_2012_1855_MOESM1_ESM.doc]

The mode of inheritance in tetraploid cut roses, C. F. S. Koning-Boucoiran et al. TAG, submitted

WUR- Plant Breeding, P.O. Box 16, 6700 AA Wageningen, The Netherlands. carole.boucoiran@wur.nl

**ESM 1:** Description of the reaction mixtures used to perform the PCR amplifications in a total volume of 20 µl

| PCR mix | 1* | 2* | 3* | 4** |  |
| --- | --- | --- | --- | --- | --- |
| Primer Forward (pmol) | 2 | 4 | 2 | 2 to 6 |  |
| Primer Reverse (pmol) | 2 | 4 | 2 | 2 to 6 |  |
| dNTP (µM each) | 625 | 625 | 1250 | 500 |  |
| *Taq* DNA polymerase (Unit) | 0.25 | 0.25 | 0.4 | 0.4 |  |
| MgCl_2_ (mM) | - | - | - | 30 |  |
| Reaction buffer (mM) |  |  |  |  |  |
| Genomic DNA | 15 | 15 | 15 | 10 |  |
|  |  |  |  |  |  |
| * Super *Taq* DNA polymerase and Super reaction buffer (Sphaero-q, The Netherlands) | | | | | |
| ** Goldstar *Taq* DNA polymerase and Goldstar reaction buffer (Eurogentec, The Netherlands) | | | | | |
